# Autocatalytic biosynthesis of abscisic acid and its synergistic action with auxin to regulate strawberry fruit ripening

**DOI:** 10.1093/hr/uhab076

**Published:** 2022-01-19

**Authors:** Tianyu Li, Zhengrong Dai, Baozhen Zeng, Jie Li, Jinyao Ouyang, Li Kang, Wei Wang, Wensuo Jia

**Affiliations:** College of Horticulture, China Agricultural University, Beijing 100193, China

## Abstract

Abscisic acid (ABA) plays a major role in the regulation of strawberry fruit ripening; however, the origin of the ABA signal is largely unknown. Here, we report an autocatalytic mechanism for ABA biosynthesis and its synergistic interaction with auxin to regulate strawberry fruit ripening. We demonstrate that ABA biosynthesis is self-induced in the achenes but not in the receptacle, resulting in its substantial accumulation during ripening. ABA was found to regulate both IAA transport and biosynthesis, thereby modulating IAA content during both early fruit growth and later fruit ripening. Taken together, these results reveal the origins of the ABA signal and demonstrate the importance of its coordinated action with IAA in the regulation of strawberry fruit development and ripening.

## Introduction

Fleshy fruits are categorized as either climacteric or non-climacteric based on physiological differences in respiratory patterns during ripening [1–4]. It is well established that the gaseous hormone ethylene is a key regulator of ripening in climacteric fruit [5–8], whereas abscisic acid (ABA) is thought to play a major role in non-climacteric fruit ripening [9–14]. At the onset of ripening in climacteric fruit, the initial biosynthesis of a small amount of ethylene triggers the rapid production of large quantities through positive feedback regulation in a process that is also referred to as autocatalytic ethylene production [15–18]. Similarly, high ABA levels accumulate during non-climacteric fruit ripening, but to date, far less is known about whether a mechanism for feedback regulation of ABA biosynthesis exists.

Strawberry has typically been classified as a non-climacteric fruit, and studies have demonstrated that ABA is of key importance in regulating its ripening [14, 19–21]. However, although studies of climacteric fruit have ascribed a primary and central role to ethylene, there is evidence that multiple phytohormones contribute to strawberry fruit ripening. Among these, indole-3-acetic acid (IAA) has been well established to be an important hormone implicated in the regulation of strawberry fruit ripening [12, 19, 22–27]. For example, in an early study, Given et al. reported that a decline in auxin concentration in the achenes during strawberry fruit maturation modulates the rate of ripening. In addition to IAA and ABA, all classes of phytohormones have been implicated in the regulation of strawberry fruit development and ripening to different degrees [22, 23, 28–31].

The involvement of multiple phytohormones implies that strawberry fruit ripening is controlled by an integrated and synergistic network, but little is known about the associated molecular mechanisms. Moreover, it is not clear whether there is a predominant central regulator analogous to ethylene in climacteric fruits. In preliminary experiments, we found no significant effect of ABA on strawberry fruit ripening when it was applied to the receptacle via injection, although fruit ripening was promoted when it was applied via fruit stalk feeding. This is consistent with a report that exogenous ABA application had no effect on strawberry fruit ripening [22]. Such results suggest that ABA may not function as the primary, or most important, signal controlling strawberry fruit ripening, but rather as a co-signal that operates in concert with other hormones. In the present study, we tested this hypothesis and investigated the integrated and synergistic action of ABA with IAA in the regulation of strawberry fruit ripening. We also demonstrated that the production of the ABA signal during ripening is an autocatalytic process. This study sheds new light on the detailed mechanisms by which phytohormones regulate the ripening of a non-climacteric fruit.

## Results

### Changes in phytohormone content during fruit development and ripening

**Figure 1:**
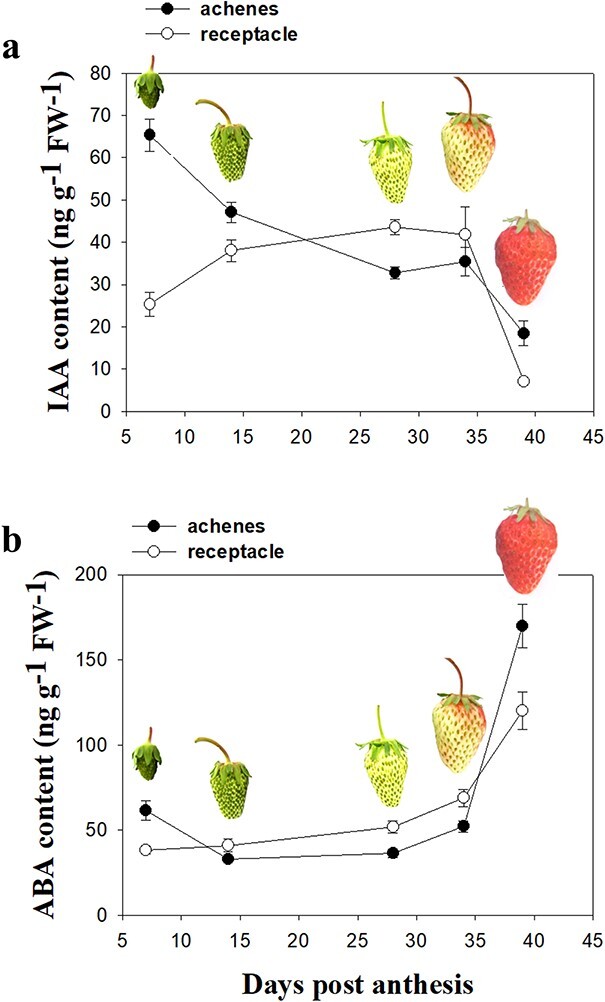
Hormone content during fruit development and ripening. **a** IAA content; **b** ABA content. Fruits were detached at different times post anthesis for hormone content measurement. For each stage, five fruits were pooled per sample for individual hormone measurements. Pictures above the data sets show fruit at the respective developmental stage. Data correspond to the mean ± SD of three biological replicates.

To understand the roles of IAA and ABA in the regulation of strawberry fruit ripening, we first examined the dynamics of their accumulation in both the achenes and the receptacle. Levels of IAA in the achenes continuously decreased from fruit set to ripening, whereas in the receptacle, they increased from fruit set through fruit growth and then decreased substantially once ripening had initiated (Fig. 1a). By contrast, the abundance of ABA in the receptacle gradually increased from fruit set and increased substantially during fruit ripening, whereas in the achenes it remained constant during early development and then increased at the onset of fruit ripening (Fig. 1b).

### The effect of exogenous ABA on strawberry fruit ripening varies depending on the application method

If ABA plays a major role in the regulation of strawberry fruit ripening, we reasoned that its exogenous application to the receptacle would promote fruit ripening. To test this idea, ABA was injected into the receptacle at a high concentration (100 μM), and its effect was analyzed using the percentage difference of phenotype (PDP) method [32] in which the effect of ABA is quantitatively evaluated using a PDP value of 0%–100%, where values <30% and >60% indicate no effect or a very strong effect on fruit ripening, respectively [32].

**Figure 2:**
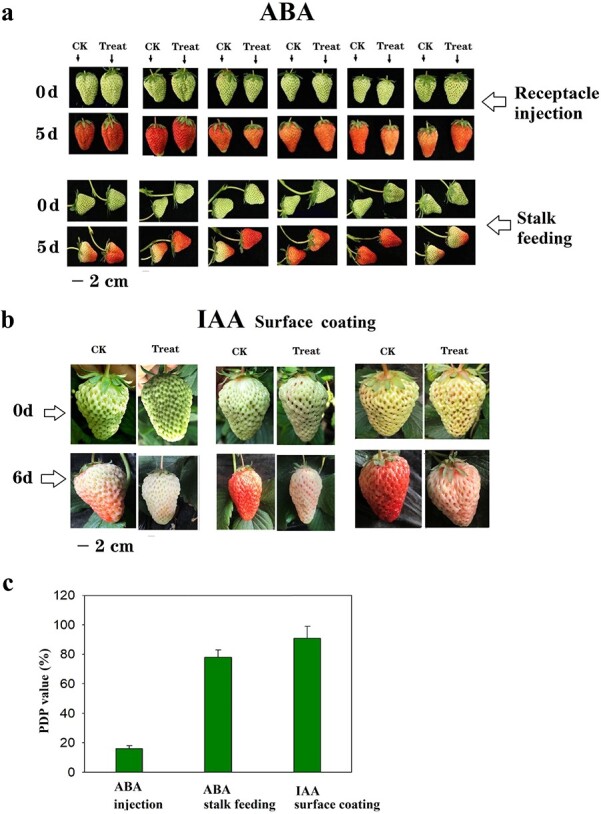
Effect of exogenous hormones on fruit ripening. **a** 100 μM ABA was injected into the receptacle or applied to the fruit via stalk feeding. Pictures were taken before the treatment (upper panel) and 5 d after the treatment (lower panel). For each treatment, 30 pairs of fruit were examined. Pictures show representative pairs of fruit. **b** 100 μM IAA was applied to the fruits via surface coating. For each treatment, 30 pairs of fruit were examined. Pictures show three representative pairs of fruit. **c** PDP analysis for the different treatments.

ABA application resulted in a PDP value of 16%, suggesting that receptacle injection of ABA was not able to substantially trigger fruit ripening (Fig. 2a, c). Intriguingly, when ABA was applied via fruit stalk feeding, fruit pigmentation was promoted, with a PDP value of 78% (Fig. 2a). Given that IAA has been shown to play an important role in the regulation of fruit ripening^33^, we next compared its effect with that of ABA. Because the transport of IAA from the achenes to the receptacle is believed to affect ripening, we applied IAA to the fruit via surface coating, and this caused a significant inhibition of fruit pigmentation, with a PDP value as high as 91% (Fig. 2b, c).

### ABA accumulation in the achenes may play a more important role than in the receptacle in ripening regulation

**Figure 3:**
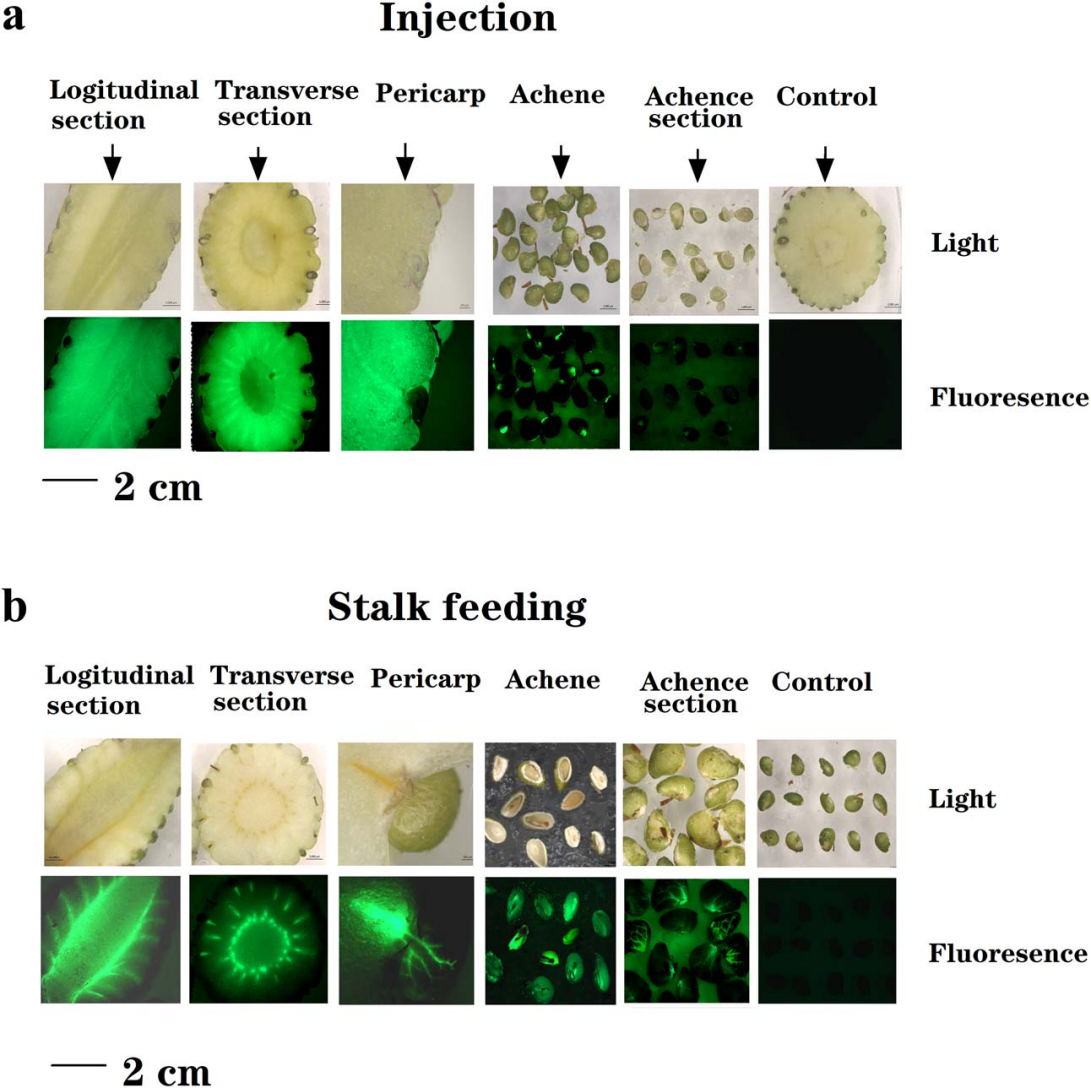
FITC fluorescence tracing experiment, showing the pattern of transport and distribution of ABA applied with different methods. **a** FITC was injected into the receptacle. Pictures were taken 1 d after the injection. **b** FITC was applied via fruit stalk feeding. Pictures were taken 1 d after the initiation of feeding. Control, injection/feeding with double-distilled water.

We hypothesized that the differing effects of ABA on ripening that resulted from the two application methods might reflect differences in ABA distribution between the achenes and the receptacle due to the method of delivery. To test this possibility, we performed a fluorescence tracing experiment to mimic the patterns of transport and ABA accumulation associated with injection and stalk feeding. FITC (3′,6′-dihydroxy-5-isothiocyanato-3H-spiro[isobenzofuran-1,9′-xanthen]-3-one), a fluorescent dye that has a similar molecular weight and polarity to ABA, was applied to the fruit via injection or stalk feeding. As shown in Fig. 3a, in the case of injection, strong fluorescence was distributed in the receptacle, whereas much less fluorescence was observed in the achenes. By contrast, following stalk feeding, the FITC was transported via the vascular bundle and accumulated in the achenes, whereas much less fluorescence appeared in the receptacle (Fig. 3b). These findings suggest that ABA introduced into the fruit via stalk feeding accumulated mainly in the achenes, whereas the injected ABA was amassed mainly in the receptacle. We therefore concluded that ABA accumulation in the achenes, rather than in the receptacle, plays a major role in ripening regulation.

### Achenes function in the regulation of receptacle ripening via IAA-mediated communication

**Figure 4:**
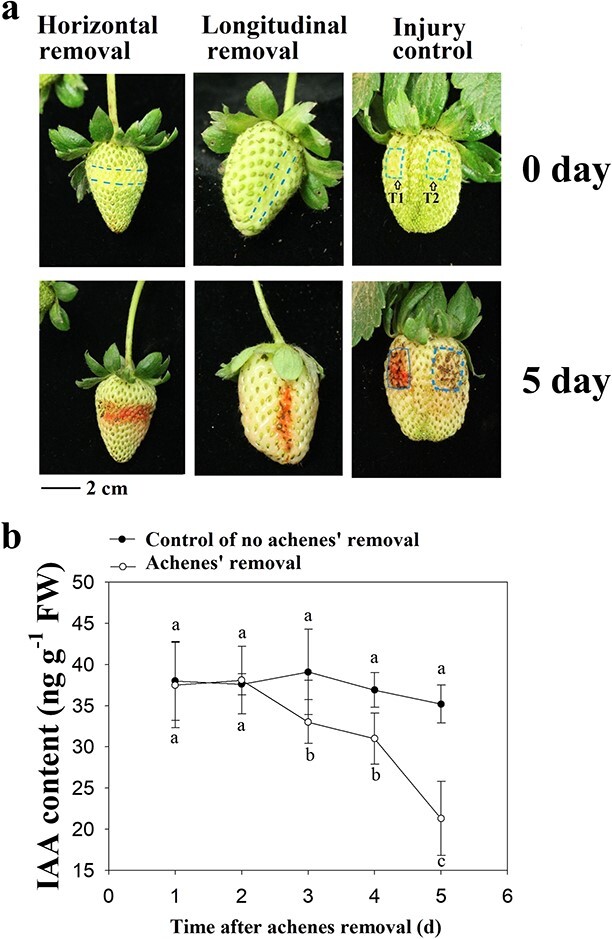
Effect of achene removal on fruit ripening in relation to the change in IAA content in the receptacle. **a** Effect of achene removal on fruit ripening. Pictures were taken immediately after achene removal (upper panel) or 5 d after removal (lower panel). T1, achenes were removed from the area framed by the dotted square; T2, achenes were injured with a needle in the area framed by the dotted square. **b** Effect of achene removal on the IAA content of the receptacle. Achenes were removed from half of each fruit, and the other half of the fruit was used as the control. IAA content was determined at different times after achene removal. Data correspond to the mean ± SD of four fruit samples. Statistically significant differences among samples were determined using Tukey’s test, and significant differences at the *P* < 0.05 level are indicated by different letters.

The above results suggest that the achenes are important for the regulation of receptacle ripening, and to provide direct evidence for this idea, we examined the effect of their removal. This resulted in rapid pigmentation of the receptacle, which we concluded was not due to physical damage, as a wounding control treatment had no effect on receptacle pigmentation (Fig. 4a). Given that IAA synthesis occurs in the achenes, we examined whether their removal affected the IAA content of the receptacle. To do this, the achenes were removed from half of a fruit at the LG stage, with the other half serving as the control. As expected, compared with the control treatment, removal of the achenes resulted in a significant decrease in the IAA content of the receptacle (Fig. 4b). Taken together, these results suggest that the achenes play a key role in the regulation of receptacle ripening via IAA export to the receptacle.

### ABA regulates the content of IAA in both achenes and receptacle

**Figure 5:**
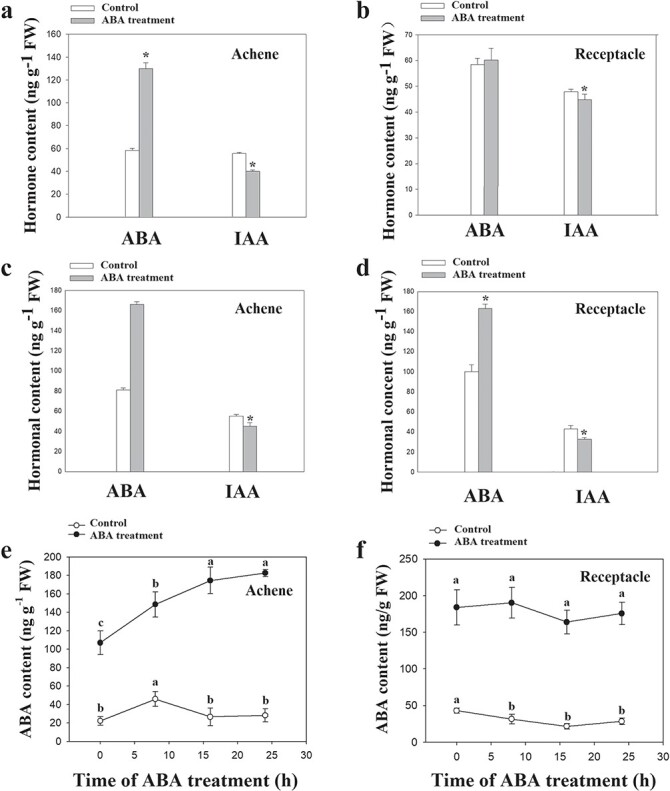
Effect of exogenous ABA treatment on endogenous ABA and IAA contents. **a** and **b**, ABA was applied via the surface-coating method. **c** and **d**, ABA was applied to the receptacles or achenes by incubating the detached achenes or receptacles in a 100 μM hormone solution for 6 h. **e** and **f**, ABA was applied to the receptacles or achenes by incubating the detached achenes or receptacles in a 100 μM hormone solution for 1 h. ABA content was measured at different time points after ABA treatment. For **a**–**d**, data correspond to the mean ± SD of three samples. ^*^P < 0.05 and ^**^P < 0.01 as determined by Tukey’s test. For **e** and **f**, significant differences at the *P* < 0.05 level are indicated by different letters.

Given the role of achene IAA in the regulation of receptacle ripening, we speculated that the effect of achene ABA on ripening might be due to its modulation of IAA transport from the achenes to the receptacle. To test this idea, we examined the effect of ABA on the content of IAA in both achenes and receptacle. The fruits were treated with exogenous ABA via surface coating (Fig. 5a, b), and the contents of ABA and IAA were measured. ABA application caused a large increase in the content of achene ABA (Fig. 5a) but not receptacle ABA. The treatment also resulted in a significant decrease in the IAA content of both achenes and receptacle. Because ABA treatment via surface coating does not cause an increase in receptacle ABA levels, the decrease in receptacle IAA content could be ascribed to decreased IAA biosynthesis or reduced transport of IAA from the achenes. We therefore tested whether ABA affects IAA content under conditions in which IAA transport is impaired. Specifically, detached achenes and receptacles were incubated with exogenous ABA (Fig. 5c, d), which caused a significant decrease in the IAA content in both achenes and receptacle, suggesting that ABA directly inhibited IAA biosynthesis. Taken together, the above results suggest that ABA affects both IAA transport and biosynthesis.

### ABA accumulation during fruit ripening is an autocatalytic process

Strawberry fruit ripening is accompanied by a large increase in ABA content in both the receptacle and the achenes, much like the ethylene burst during climacteric fruit ripening (Fig. 1b). The climacteric fruit ripening-associated ethylene burst results from autocatalytic ethylene production, and we hypothesized that ABA accumulation during strawberry fruit ripening might be similarly autocatalytic. To test this possibility, detached receptacles and achenes were separately incubated with exogenous ABA for 1 h, and then the endogenous ABA content was measured. As shown in Fig. 5e, in the achenes, ABA treatment caused a steady increase in the levels of endogenous ABA following the treatment, whereas endogenous ABA levels were unchanged in the untreated control. In the receptacle, although treatment with exogenous ABA caused an increase in the levels of ABA, the elevated level of ABA remained unchanged following the treatment (Fig. 5f). This observation suggests that the ABA accumulation associated with fruit ripening in the achenes is an autocatalytic process.

### The expression of ABA biosynthesis genes is induced by ABA in the achenes

**Figure 6:**
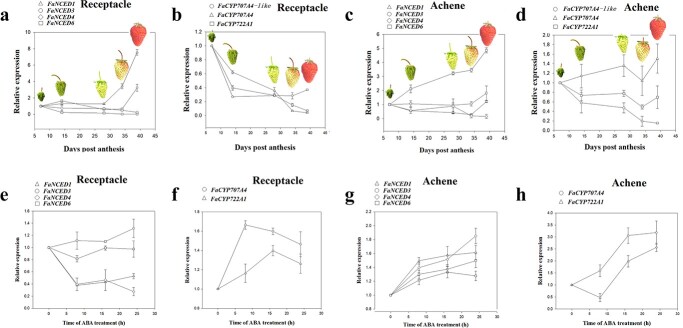
Expression of genes involved in ABA biosynthesis and catabolism in relation to fruit development and ABA treatment. **a**–**d** Gene expression patterns through fruit development and ripening. **a** and **c**, ABA biosynthesis genes. **b** and **d**, ABA catabolism genes. Gene expression was analyzed by qRT-PCR. **e**–**h** Gene expression in response to exogenous ABA treatment. **e** and **g**, ABA biosynthesis genes. **f** and **h**, ABA catabolism genes. *FaACTIN* was used as a control gene for expression normalization. Values correspond to the mean ± SD of five fruits.

Dynamic changes in ABA content are determined by ABA biosynthesis and catabolism, and 9-cis-epoxycarotenoid dioxygenase (NCED) and 8′-hydroxylase are well established as key enzymes that control ABA biosynthesis and catabolism, respectively. Bioinformatic analysis identified four *NCED* genes (*FaNCED1*, *FaNCED3*, *FaNCED4*, and *FaNCED6*) and three genes encoding putative ABA 8′-hydroxylases (*FaCYP707A4*, *FaCYP707A4-*like, and *FaCYP722A1*) (Fig. S1). To elucidate the molecular basis for the change in ABA content, we examined the expression patterns of these genes throughout fruit development and ripening (Fig. 6a–d). Among the four *NCED* genes expressed in the receptacle, *FaNCED1* and *FaNCED3* were substantially upregulated during ripening (Fig. 6a), whereas all the 8′-hydroxylase genes were significantly downregulated during the whole developmental process (Fig. 6b). This suggests that in addition to the induction of the *NCED* genes, the downregulation of the 8′-hydroxylase genes also contributes to fruit development–associated ABA accumulation. Notably, in the achenes, the expression of *FaNCED6,* rather than *FaNCED1* and *FaNCED3*, was greatly induced, and its expression remained high from early to late ripening stages (Fig. 6c).

Given the apparent autocatalytic ABA production in the achenes (Fig. 6), we examined whether the *FaNCED* genes might be upregulated in response to ABA stimulus. As expected, all four were upregulated in response to ABA treatment (Fig. 6g). By contrast, in the receptacle, ABA treatment resulted in a decrease, rather than an increase, in the expression of two genes (*FaNCED1* and *FaNCED4*), whereas the expression of the other two genes was not significantly affected. These observations further indicated that the ABA accumulation is a result of autocatalytic ABA production in the achenes but not in the receptacle. Notably, in both achenes and receptacle, expression of the 8′-hydroxylase genes was induced by ABA treatment (Fig. 6f, h). These results provide further evidence that the ABA accumulation in the achenes results from autocatalytic ABA biosynthesis.

### Key genes respond positively to ABA to transport IAA to the achenes and negatively to induce IAA biosynthesis in the receptacle

**Figure 7:**
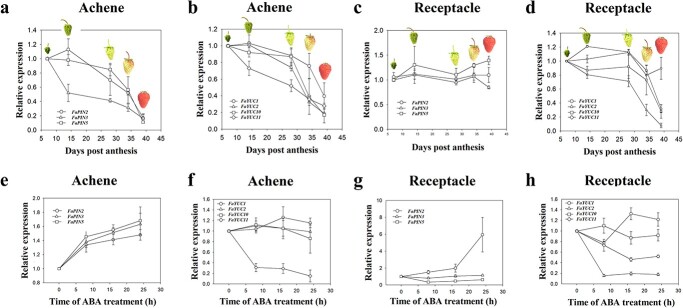
Expression of genes involved in IAA transport and biosynthesis in relation to fruit development and ABA treatment. **a**–**d** Gene expression during fruit development and ripening. **a** and **c**, IAA biosynthesis genes. **b** and **d**, IAA catabolism genes. Gene expression was analyzed by qRT-PCR. **e**–**h** Gene expression in response to exogenous ABA treatment. **e** and **g**, IAA biosynthesis genes. **f** and **h**, IAA catabolism genes. *FaACTIN* was used as a control gene for normalization. Values correspond to the mean ± SD of five fruits.

To characterize the role of ABA in regulating IAA content, we examined the expression of key IAA transport and biosynthesis genes. Bioinformatic analysis identified five genes involved in IAA transport, designated *FaPIN1*–*5* (Fig. S2), as well as nine genes implicated in the last step of IAA biosynthesis, designated *FaYUC1*, *2*, *3*, *4, 6*, *7*, *8*, *10*, and *11* (Fig. S3). Reverse transcription (RT)-PCR analysis identified three *PIN* genes (*FaPIN2*, *FaPIN3*, and *FaPIN5*; Fig. S4a) and four *YUC* genes (*FaYUC1*, *FaYUC2*, *FaYUC10*, and *FaYUC11*; Fig. S4b) that were highly expressed in the achenes and receptacle. Notably, the expression levels of nearly all these genes were much higher in the achenes than in the receptacle, suggesting that IAA transport and biosynthesis are more active there. Quantitative (q)RT-PCR analysis indicated that the expression of all the IAA transport and biosynthesis genes decreased greatly through fruit development and ripening in the achenes (Fig. 7a, b). By contrast, in the receptacle, the expression of the *FaPIN* genes showed little variation (Fig. 7c), whereas the expression of nearly all the *FaYUC* genes decreased during ripening (Fig. 7d).

Fig. 7e–h shows the patterns of gene expression in response to ABA stimulus. In the achenes, the expression of all the genes involved in IAA transport was greatly promoted by the ABA treatment (Fig. 7e), consistent with the idea that ABA enhances IAA transport from the achenes to the receptacle. In the receptacle, however, the three *FaPIN* genes showed different responses to ABA treatment: ABA treatment caused a significant increase in *FaPIN2* expression and a decrease in *FaPIN5* expression, but it had no effect on *FaPIN3* expression. *FaYUC11* expression was arrested by ABA in the achenes (Fig. 7f). Importantly, both *FaYUC10* and *FaYUC11*, two highly expressed genes, were greatly inhibited by ABA in the receptacle (Fig. 7h), suggesting that ABA inhibits IAA biosynthesis in the receptacle.

## Discussion

### ABA accumulation during fruit ripening is an autocatalytic ABA biosynthetic process

Plant cell division and differentiation are tightly controlled by the dynamic equilibrium of multiple phytohormones, and the level of each phytohormone is therefore tightly controlled. One of the important mechanisms for this control is feedback regulation of phytohormone biosynthesis, in which positive and negative feedback systems act to increase and decrease hormone levels. In climacteric fruits, ethylene accumulation during fruit ripening is regulated by positive feedback, or autocatalytic biosynthesis, in which an initially small amount of phytohormone triggers its own massive production.

As with the ethylene accumulation pattern in climacteric fruits, ABA also accumulates to high levels during ripening in non-climacteric fruit. In the present study, we found that ABA accumulation was more substantial in the achenes than in the receptacle in the later fruit ripening stage, as indicated by higher ABA content in the achenes than in the receptacle in the R stage and lower content in the achenes than in the receptacle before the W stage. To investigate whether ABA accumulated through an autocatalytic process, fruit samples were transiently treated with ABA, after which ABA content was continuously monitored for 24 h. We reasoned that if the rate of ABA biosynthesis was constant or decreased, ABA content should remain unchanged or decrease due to ABA catabolism. Strikingly, transient ABA treatment caused a continuous increase in the ABA content of the achenes (Fig. 5b) but not of the receptacle (Fig. 5a). We concluded that the transient ABA treatment triggered a major increase in the rate of ABA biosynthesis in the achenes due to autocatalytic ABA biosynthesis. This discovery reveals a common mechanism for the origin of key ripening-associated phytohormone signals between climacteric and non-climacteric fruits. Future investigation of the mechanism regulating ABA autocatalytic production will further contribute to our understanding of the regulation of non-climacteric fruit ripening.

### Does ABA have a major role in the regulation of strawberry fruit ripening?

It has been suggested that ABA plays a major role in regulating the ripening of non-climacteric fruit [14, 19–21], including strawberry. An important question, however, is the relative predominance of its role compared with the action of other hormones. Ethylene is undoubtedly the “major phytohormone” controlling climacteric fruit ripening, as small amounts of exogenous ethylene can trigger the ripening cascade. In the present study, we did not find that application of ABA to the receptacle via injection strongly promoted fruit ripening. A previous study [22] also indicated that exogenous ABA treatment had no significant effect on strawberry fruit ripening. Accordingly, whether the receptacle-originated ABA signal can be regarded as a primary signal deserves further investigation.

A decline in IAA content in the receptacle is known to contribute to promoting strawberry fruit ripening, and IAA thus functions as a negative signal. It has also been established that IAA is mainly synthesized in the achenes [33–37], and in the present study, we found that removal of the achenes promoted receptacle pigmentation (Fig. 4a). Notably, this effect was much stronger than that caused by any of the other phytohormones tested. While it is not possible to say that IAA plays a more important role than ABA in terms of their effects on receptacle pigmentation, the manipulation of IAA content affected receptacle pigmentation more strongly than the manipulation of ABA content (Fig. 2a, b). Given these results, we conclude that ABA does not act as a major, or central, phytohormone in the control of strawberry fruit ripening.

### Roles of IAA and ABA in relation to the origins of fruit organs

Although strawberry is considered to be a canonical non-climacteric fruit, it should be noted that it is distinct from most other non-climacteric fruits in terms of its developmental origins. The edible part of the strawberry originates from the receptacle, such that strawberry is a typical spurious fruit, with the achenes being the true fruit. In the present study, we demonstrated that ABA only applied to the achenes was able to substantially promote fruit ripening (Fig. 2a, 2b and Fig. 3).

Importantly, we demonstrated that the ABA accumulation during fruit ripening was due to autocatalytic biosynthesis but that this occurs only in the achenes and not in the receptacle. This finding highlights the differences between achenes and receptacles as distinct organs. Based on both theoretical inference and experimental evidence, we propose that ABA plays a major role in the ripening regulation of the achenes rather than the receptacle.

In nature, fleshy fruit ripening is aimed at promoting seed dispersal [38, 39], and therefore, the process of seed ripening should be tightly integrated with ripening of the fleshy parts. This integration between the achenes and the receptacle is achieved by signal communication between the two. If ABA acts as a central signal controlling achene ripening, it is therefore likely to play an important role in the regulation of receptacle ripening. Alternatively, the role of ABA in the regulation of receptacle ripening may be largely ascribed to its regulation of achene ripening, although its direct role in the regulation of receptacle ripening is also a part of the whole fruit ripening process. IAA is well known for its primary role in growth promotion of vegetative organs. As the strawberry receptacle originates from a vegetative tissue/organ, it would be expected to be regulated primarily by IAA. Accordingly, we propose that the action of ABA in strawberry fruit ripening is strongly influenced by its regulation of IAA biosynthesis and transport.

### Mechanisms for the integration and synergistic action of IAA and ABA

In the present study, we provide both physiological and biological evidence that IAA signal communication is linked to ABA action and that this interaction is important for the control of early fruit development and later ripening. Recently, we demonstrated that strawberry fruit ripening is initiated by cell separation, and importantly, that the cell separation is initiated from fruit set, suggesting that the initiation of fruit ripening is determined by cellular processes that occur during early fruit growth and development [40–43]. IAA regulates cell growth and plays a primary role in the regulation of early fruit growth and development. Consistent with this notion, IAA content was found to be unchanged or even to increase during development until the onset of fruit ripening at the “W” stage and then to decrease after the onset of fruit ripening. We conclude that IAA plays dual roles in the regulation of strawberry fruit development and ripening. First, in the early stages, it acts to promote fruit growth and expansion, and second, in the later stages, it acts to initiate fruit ripening by mediating a release from ripening inhibition.

To further demonstrate that ABA regulates IAA content, we examined the effect of ABA on the expression of genes encoding IAA transport and biosynthesis proteins. RT-PCR analysis indicated that the transcript levels of all the investigated genes were much higher in the achenes than in the receptacle (Fig. S4). This is consistent with the observation that the IAA content was significantly higher in the achenes than in the receptacle in the early developmental stage. However, although IAA content declined from fruit set to ripening, it increased before the onset of fruit ripening (Fig. 1a). Because the expression of the IAA biosynthesis genes decreased at this time, we conclude that IAA transport from the achenes to the receptacle contributes greatly to the increase in IAA content in the receptacle. Importantly, IAA abundance decreased substantially from the onset of fruit ripening. If IAA transport from the achenes contributes to the observed large increase in IAA content in the receptacle, it is difficult to understand how the IAA content decreases during ripening. To address this question, we examined the achenes to receptacle biomass ratio, which clearly decreased during fruit development and ripening (Fig. S5) in parallel with a decline in IAA content. Collectively, these results suggest that the contribution of IAA from the achenes to the receptacle may be neglected, such that, the decrease in IAA content reflects the rate of IAA biosynthesis in the receptacle. Accordingly, the expression of IAA biosynthesis genes was found to be arrested by ABA treatment, suggesting that the decline in IAA levels results from ABA accumulation during fruit ripening.

**Figure 8:**
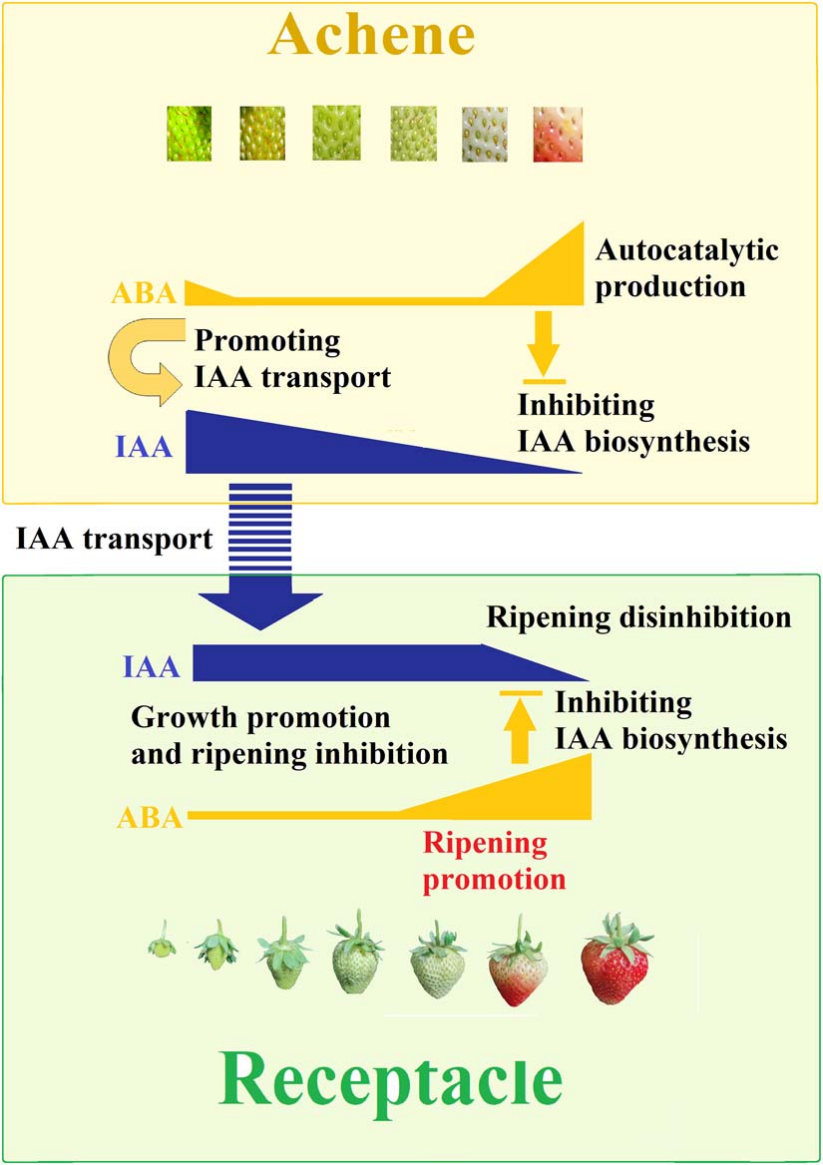
Model of the synergistic action of IAA and ABA in the regulation of strawberry fruit ripening. Early fruit growth and expansion lays the foundation for fruit ripening, whereas their inhibition at the later stage is necessary for the initiation of fruit ripening. IAA is primarily synthesized in the achenes, and its transport from the achenes to the receptacle is necessary for receptacle growth and expansion. A major reduction in IAA content initiates receptacle ripening. ABA acts to promote IAA transport from the achenes to the receptacle at the early stages, and it inhibits IAA biosynthesis at the later stages, such that a large increase in ABA levels due to autocatalytic production promotes receptacle ripening.

In summary (Fig. 8), IAA levels in the achenes decrease during fruit development and ripening but only start to decrease at the onset of fruit ripening in the receptacle. The high level of IAA in the receptacle in the early stages can largely be ascribed to IAA transport from the achenes to the receptacle, and the substantial decline in IAA during fruit ripening results from an inhibition of IAA biosynthesis in the receptacle. The large increase in ABA abundance results from positive feedback regulation of ABA biosynthesis, or autocatalytic ABA production. ABA in the achenes acts to promote IAA transport from the achenes to the receptacle at an early developmental stage, thereby keeping IAA content constant and promoting fruit growth and expansion. The major accumulation of ABA in the receptacle acts to inhibit IAA biosynthesis and thus leads to a decrease in IAA content, thereby relieving the inhibition of ripening by IAA.

## Materials and methods

### Plant materials and growth conditions

*Fragaria × ananassa* Duch. ‘Benihoppe’ was used in this research. Plants were grown under conditions of 18–28°C with an 8-h/16-h dark/light cycle. Plants were well watered and free from abiotic and biotic stress. At different stages, attached or detached fruits were used, depending on the specific aims of the different experiments as described below.

### Reagents

IAA, ABA, JA, and FITC were purchased from Sigma-Aldrich (USA). Agar was purchased from Oxoid (UK). The RNA extraction kit was purchased from Sigma (USA). The RNA reverse transcription kit was purchased from Novoprotein (Shanghai, China). The qRT-PCR kit was purchased from Biomarker (Beijing, China).

The main buffers used were a 100 μM ABA solution and a 100 μM IAA solution, which were both prepared immediately prior to use.

### Pharmacological experiments

Three methods were used to apply phytohormones to strawberry fruit: injection, stalk feeding, and surface coating. For all experiments, ABA and IAA were used (100 μM each), and fruit pairs were observed in which one fruit was treated with hormone and the other with double-distilled water as a control. Specifically, fruits were detached at the mid-sized green fruit (MG) stage and paired based on color, size, and shape. For the injection treatment, the syringe needle was introduced into the stem end of the fruit and pushed into the fruit center, and the hormone was then injected slowly until the whole fruit was fully infiltrated. The fruits were then allowed to recover to their original weight (i.e. allowing the hormone solution to evaporate in case of hypoxia stress) under ambient conditions (22°C) and then incubated for 5 d at 100% humidity. For the feeding experiment, fruits were detached by cutting with a sharp razor blade from the base of the fruit stalk. The stalks were immediately inserted into the hormone solution or double-distilled water, and the fruits were then incubated for 5 d at 22°C. The surface-coating method was used for the IAA and ABA treatments. IAA or ABA was dissolved in 0.2% agar at 22°C. Surface coating was achieved by dipping fruits briefly into the IAA-agar or ABA-agar solutions, such that IAA or ABA evenly covered the fruit surface. The effect of the hormones was quantitatively evaluated by the PDP method as described below.

### Percentage difference of phenotype (PDP) analysis

The PDP method can be used to quantitatively evaluate the developmental difference between two groups of fruit^32^. Fruits were paired based on their developmental stage and phenotypes, such as color, size, shape, and swelling status of the receptacle. For each pair, one fruit was used for hormone treatment, and the other was treated with double-distilled water to serve as a control. After the hormone treatment, pigment accumulation was examined until one of the paired fruits started to redden. The reddened fruits were marked “1”, whereas the non-reddened fruits were marked “0”. The percentage of fruits marked “1” relative to the total number of fruit pairs was calculated. The percentage difference between the hormone treatment and the non-treatment control was designated as the PDP value, which could vary from 0 to 100%.

### Fluorescence tracing experiment

Fluorescence tracing was performed to observe the patterns of ABA transport and distribution using the different pharmacological methods. FITC, a fluorescent dye that has a similar molecular weight and polarity to ABA, was used for the tracing experiment. FITC was applied to the fruits as described for ABA, above. Fluorescence imaging was performed using a homemade apparatus with an argon laser, a 488-nm excitation filter, and a 507-nm emission filter for analyzing and imaging objects up to 100 cm^2^ in surface area.

### Hormone quantification

Hormone measurements were performed according to previously published methods [44]. In brief, fruits were frozen in liquid nitrogen, and five fruits were pooled as a single sample. Fruit samples were ground into homogenate, then extracted overnight at 4°C in 80% methanol. The extract was centrifuged at 10 000 rpm for 40 min to remove cell debris. The supernatant was evaporated under vacuum to remove the methanol, and the pellet was redissolved in 1 M aqueous formic acid. IAA and ABA were eluted with methanol and dried under vacuum. The residue was redissolved in 1 M formic acid and loaded onto a preactivated, 3-cc Oasis cation MAX SPE cartridge (Waters). After washing with 1 M formic acid, 0.1 M ammonium hydroxide, IAA, and ABA were combined, dried under vacuum, and redissolved in high-pressure liquid chromatography (HPLC) initial mobile phase. The hormones were measured with an HPLC (SHIMADZU) system using an autosampler in no-waste mode.

### Removal of achenes

Achenes were removed from fruits at the LG stage with a fine needle. A control experiment was performed to rule out a potential effect of injury caused by achene removal. Fruits were wounded with a needle to the same degree as that occurred during achene removal. To examine the effect of achene removal on the IAA content of the receptacle, the achenes were removed from half of a fruit with a needle, and the other half of the fruit was used as a control. IAA content was examined at different times after achene removal.

### RT-PCR and qRT-PCR

RT-PCR and qRT-PCR were performed to examine the expression of genes related to the regulation of ABA and IAA biosynthesis and catabolism. Primer sequences used for RT-PCR and qRT-PCR are shown in Table S1 and Table S2, respectively. Samples were powdered in liquid nitrogen, and total RNA was isolated using an E.Z.N.A. RNA Kit (Omega Bio-Tek). cDNA was synthesized using the reverse transcription kit NovoScript Plus All-in-One 1st Strand cDNA Synthesis Supermix (Novoprotein) according to the manufacturer’s instructions. qRT-PCR was performed using SYBR Premix Ex Taq (Biomarker) with an ABI QuantStudio 6 Real-Time PCR System (Applied Biosystems). qRT-PCR primers were designed using an NCBI tool (https://www.ncbi.nlm.nih.gov/tools/primer-blast/index.cgi?LINK_LOC=BlastHome). Each sample was analyzed in triplicate. Gene22626 (*FaACTIN*) was used as an internal control, and the 2^−ΔΔCt^ method was used to determine transcript levels.

### Statistical analysis

For each treatment, five individual fruits were pooled to constitute one individual sample, and measurements were conducted in triplicate. Student’s *t*-tests were used to test the statistical significance of treatment differences, with *P* < 0.05 deemed to be significant.

## Acknowledgements

This work is financially supported by the National Key Research and Development Program (2019YFD1000200), the National Natural Science Foundation of China (31872086), the National Key Research and Development Program (2019YFD1000800), and the Construction of Beijing Science and Technology Innovation and Service Capacity in Top Subjects (CEFF-PXM2019_014207_000032).

## Author contributions

W.J. designed the research and prepared the paper. T.L., Z.D., B.Z., and T.L. performed the experiments. O.J., J.L., K.L., and W.W. participated in some of the experiments.

## Data availability

All data supporting these research results can be obtained from the paper and its Supplementary Materials published online.

## Conflict of interest statement

The authors declare no competing interests.

## Supplementary data

Supplementary data is available at *Horticulture Research Journal* online.
